# Neuronal progenitors of the dentate gyrus express the SARS-CoV-2 cell receptor during migration in the developing human hippocampus

**DOI:** 10.1007/s00018-023-04787-8

**Published:** 2023-05-07

**Authors:** José Manuel Hernandez-Lopez, Cristina Hernandez-Medina, Cristina Medina-Corvalan, Mónica Rodenas, Almagro Francisca, Claudia Perez-Garcia, Diego Echevarria, Francisco Carratala, Emilio Geijo-Barrientos, Salvador Martinez

**Affiliations:** 1 Instituto de Neurociencias UMH-CSIC, Avda. Ramon y Cajal sn, 03550 San Juan de Alicante, Spain; 2 Cátedra de Neurosciencia, UCAM-San Antonio, Murcia, Spain; 3 Department of Anatomy, Univ. Murcia, Murcia, Spain; 4 Pediatric Service Univ. Hospital San Juan, San Juan de Alicante, Spain; 5 Center of Biomedical Network Research on Mental Health (CIBERSAM), ISCIII, Madrid, Spain

**Keywords:** COVID-19, ACE2, Brain viral infection, Dentate gyrus, SARS-CoV-2, Neurotropism

## Abstract

The COVID-19 pandemic spread around the world is due to the enormous capacity of the SARS-CoV-2 coronavirus to be transmitted between humans, causing a threat to global public health. It has been shown that the entry of this virus into cells is highly facilitated by the presence of angiotensin-converting enzyme 2 (ACE2) in the cell membrane. Currently, we have no precise knowledge of how this receptor expresses in the brain of human fetus and, as a consequence, we do not know how susceptible the neural cells in the developing brain are to being infected through the vertical transmission of this virus, from mother to fetus. In this work, we describe the expression of ACE2 in the human brain at 20 weeks of gestation. This stage corresponds to the period of neuronal generation, migration, and differentiation in the cerebral cortex. We describe the specific expression of ACE2 in neuronal precursors and migratory neuroblasts of the dentate gyrus in the hippocampus. This finding implies that SARS-CoV-2 infection during the fetal period may affect neuronal progenitor cells and alter the normal development of the brain region where memory engrams are generated. Thus, although vertical transmission of SARS-CoV-2 infection was reported in few cases, the massive infection rate of young people in terms of the new variants leads to the possibility of increasing the ratio of congenital infections and originating cognitive alterations, as well as neuronal circuit anomalies that may represent vulnerability to mental problems throughout life.

**Supplementary Information:**

The online version contains supplementary material available at 10.1007/s00018-023-04787-8.

## Introduction

The worldwide COVID-19 pandemic, with infection detected in more than 700 million humans and more than 6.8 million deaths (WHO, global pandemic situation in March 2023), represents the most important challenge ever to human health. To understand the impact of the SARS-CoV-2 coronavirus infection in patients, it is necessary to understand the vulnerability of the organs and tissues that are infected [1]. The organotropism of SARS-CoV-2 beyond the respiratory tract may have some influence in the course of the disease, aggravating pre-existing conditions, and it is necessary to predict the consequences of the viral infection on the entire organism as well as in different social groups. Especially important are those people at risk of developing a serious disease and potentially dying, but also those in which the consequences of the infection may compromise their future life. New viral variants increase viral transmission, especially in the non-vaccinated population, but also among the vaccinated; the former group includes predominantly young people and the population of underdeveloped countries. Since the possibility of infection and pregnancy is high in these particular human populations, the possibility of the virus being transmitted vertically to their descendants makes it necessary to explore fetal vulnerability to SARS-CoV-2 infection. Very few data have been published on maternal–fetal transmission, although placental infection and viral transmission to the fetus have been reported [2, 3]. Fetal infection has been described by Vivanti et al. [4] resulting in severe inflammatory anomalies in the brain neurovascular unit, including gliosis and vascular microinfarcts, which are similar to the alterations reported in adult brain infections [5–7]. Although blood-borne transmission is possible since viremia is detectable in symptomatic adults [8], the mechanisms of viral transmission from an infected mother to a fetus are not well known [9, 10]; [reviewed by [11]. There is a general consensus about the need for improved studies on the fetal consequences of maternal SARS-CoV-2 infection, especially because of the high incidence of viral transmission among the fertile population, and the fact that the currently approved vaccines do not prevent transmission. Moreover, potential mutations of SARS-CoV-2 may represent increased viral infectivity, including maternofetal transmission. Recently, a report by Piekos et al. [12] showed that preterm delivery and stillbirth are related to SARS-CoV-2 infection during pregnancy.

Fetal brain infection may trigger a strong neuroinflammatory process, like in the parenchyma of the lung, with severe microvascular inflammation [4]. However, even without the development of strong vascular inflammatory-associated anomalies, coronavirus infection in developing neural cells could produce primary neural phenotypes by altering normal neuronal differentiation and maturation, generating pathogenetic consequences that may represent anomalies in mental function throughout life [13, 14].

It has been shown that SARS-CoV-2 entry into cells is greatly facilitated by the presence of the angiotensin-converting enzyme ACE2 in the cell membrane [15]. Cells expressing ACE2 are potential SARS-CoV-2 targets and suffer coronavirus infection that may modify cell function directedly, by viral cytotoxicity, and undirectedly, related to the disruption of the trophic effects of renin–angiotensin system activation [16, 17]. ACE2 expression in the adult brain has been detected in multiple regions, being expressed in the amygdala, pons and medulla; while the highest level of expression is in the temporal lobe cortex [5]. Matshke et al. [6] reported the expression of SARS-CoV-2 receptors, including ACE2, in the neurons and oligodendrocytes of COVID-19 patients. Song et al. [18] demonstrated that ACE2 is required for SARS-CoV2 infection in human brain organoids. It is therefore urgent to determine the expression of ACE2 in fetal brain cells in order to recognize the neurological targets in the developing brain and adequately manage pregnant women to prevent viral infection, as well as to monitor the brain maturation and mental health of potentially affected children.

To date, studies have reported ACE2 expression in the embryonic brain through transcriptomic RNA sequencing in preimplantation stages [19, 20], but only Faure-Bardon et al. [21] have provided evidence of the ACE2 protein in human fetal brain cells, reporting ACE2 expression in the choroidal plexus of a 21-post-gestational-week fetus. The presence of ACE2 in choroidal plexus epithelium cells suggests the possibility of viral dissemination into the ventricular system and the subsequent infection of the neural parenchyma through ventricular dissemination [11]. Infection of an immature choroidal plexus with increased blood–brain barrier (BBB) permeability may represent a pathway for infecting the cerebrospinal fluid (CSF), and through the ventricular ependymal layer the infection may reach the subventricular zone infecting neural precursors. Moreover, from the CSF, parenchymal infections are possible through cisternal Virchow-Robins spaces, thereby infecting perivascular cells (pericytes and astrocytes). In addition, once the fetus becomes infected, the coronavirus can reach the neural parenchyma via the vascular system, since its immature BBB is not an adequate defense against viral invasion. Neural cells with ACE2 receptors may then become infected. Direct neuronal damage in COVID-19 has been demonstrated in human brain organoids, and it has been reported that SARS-CoV-2 may infect neurons and cause neuropathological symptoms [18, 22], reviewed by 23].

Brain function depends on neural structural complexity, which results from the precise articulation of specific developmental processes, i.e., neurogenesis, neural migrations, differentiation, and synaptogenesis. Several types of viral infections during pregnancy have been described as teratogenic factors that disrupt these developmental processes, generating structural brain anomalies, epilepsy, and mental diseases [reviewed by 24]. In light of the current exposure of the global population to SARS-CoV-2, and its increasing infectiveness due to new variants, it is critical to map the brain regions that may be potentially infected, to determine the cellular targets and developmental processes that could be affected in the fetus.

## Materials and methods

We studied the expression of ACE2 in three human brains at 20 weeks of gestation. The fetuses were obtained from anonymous donations after spontaneous abortion, from the Anatomy Innovation Service and the Histology and Anatomy Department of Miguel Hernandez University Medical School. The data reproduced in this work utilizes human tissue that was procured via the Anatomy Innovation Service, which provides de-identified samples. The study was reviewed and deemed exempt from ethical concerns by our Institutional Ethical Review Board. The Anatomy Collection maintenance protocols are in line with the ethical standards of our institution and with the 1964 Helsinki declaration and its later amendments or comparable ethical standards. After reception, the fetuses were identified using an anonymous collection code and classified and fixed for at least 3 months by immersion in 10% formalin after the removal of the skin over the cranial vault. The brain was then extracted and post-fixed for two weeks in 4% paraformaldehyde in phosphate buffer (pH 7.4 0.1 M) at 4 ºC. Then, each hemisphere was subdivided into 5 blocks for paraffin inclusion and sectioning in the coronal plane, at 7–10 microns thickness. Selected slides from the collection were stained with Cresyl violet for use in practical neuroanatomy courses. The remaining sections were conserved. From this collection, selected slides in a parallel series were used for this study. We have also processed adult human hippocampus by ACE2 immunohistochemistry in paraffin included sections, cut at 7 microns thick, from human brain sections collection of the Anatomy Innovation Service.

### Cell culture

Human premolars were extracted and collected from normal adult patients undergoing orthodontic therapy in the University Hospital. Before extraction, the patients were informed of the procedures to be performed and signed a written consent. The procedure was reviewed and deemed exempt from ethical concerns by our Institutional Ethical Review Board. Dental pieces were scraped from the middle third region of the root surface. After washing the extracted periodontal ligament with Ca^++^ and Mg^++^-free Hank's balance salt solution (HBSS; Gibco, Gaithersburg, MD, USA), it was digested with 3 mg/ml type I collagenase (Worthington Biochemical Corporation, Lakewood, NJ, USA) and 4 mg/ml dispase II (Gibco) in alpha modification minimum essential medium eagle (α-MEM; Sigma-Aldrich, St. Louis, MO, USA) for 1 h at 37 °C. The reaction was stopped by the addition of α-MEM. The dissociated tissue was passed through a 70-μm cell strainer (BD Falcon, Bedford, MA, USA). Cells were centrifuged, and the pellet was resuspended in serum-containing media (designated as the basal media), composed of α-MEM supplemented with 10% calf serum (Sigma), 100 units/ml penicillin–streptomycin (Sigma), 50 mg/ml l-ascorbic acid (Sigma), and 2 mM l-glutamine (Sigma). The cell suspension was plated into six-well multiwell plates (BD Falcon) and incubated at 37 °C in 5% CO_2_.

### Immunohistochemistry

The immunohistochemistry was performed on 200 sections of each fetal brain and 20 sections of adult hippocampus using as primary antibodies: rabbit polyclonal anti-ACE2 (Abcam Cat# ab15348, RRID:AB_301861; 1/500); rabbit polyclonal anti-ACE2 (Sigma-Aldrich Cat#HPA000288, RRID:AB_1078160; 1/500), mouse monoclonal anti-ACE2 (R&D Systems Cat# MAB933, RRID:AB_2223153; 1/500), rat monoclonal anti-GFAP (Millipore Cat# 345,860-100UG, RRID:AB_10685458; 1/200); mouse monoclonal anti-Neuropilin-1 (Invitrogen Cat# 14-3042-82, RRID:AB_2572873**;** 1/200**),** goat polyclonal anti-Doublecortin (Santa Cruz Biotechnology Cat# sc-8066**,** RRID:AB_2088494; 1/100); and rabbit polyclonal anti-TBR2 (Abcam Cat# ab23345, RRID:AB_778267; 1/200). For the immunohistochemistry, the paraffin was first removed using xylene, and the sections were rehydrated in decreasing ethanol concentrations in phosphate-buffered saline (PBS; 0.1 M, Ph7.4). Next, the sections were treated with citrate buffer (pH 6.0), as a heat-induced antigen retriever prior to the application of antibodies, after incubation in 0.3% H_2_O_2_ in PBS-T (PBS + 1% Triton X-100) for 30 min at room temperature to remove endogenous peroxidase activity. The slides were then incubated in 1% Triton X-100, 1% BSA, and Lysine 0.1 M in PBS for 1 h at room temperature (RT) on a rocking table. They were then incubated in primary antibodies diluted in Vision FLEX Antibody Diluent (DAKO, Denmark) for 48 h (RT). The sections were rinsed three times in PBS at RT and incubated overnight in the corresponding biotinylated secondary antibody at the producer’s recommended concentrations, diluted in Vision FLEX Antibody Diluent (DAKO, Denmark). Afterward, the sections were washed with PBS-T and incubated with Avidin–Biotin Complex for 3 h (1:300; ABC kit, Vector Laboratories CA-94010) diluted in 1% Triton X-100 and Lysine 0.1 M in PBS. For single colorimetric detection (brown), the tissue was incubated with 1% 3,39-Diaminobenzidine (DAB; Vector Laboratories SK-4100), and 0.0018% H_2_O_2_ in PBS. For double colorimetric detection, we first developed the selected primary antibody in black, incubating with 1% 3,39-Diaminobenzidine (DAB; Vector Laboratories Cat# SK-4100**,** RRID: AB_2336382, 0.025% ammonium nickel sulfate hexahydrate, and 0.0018% H_2_O_2_ in PBS). Then, we initiated the second immunostaining with the selected antibody to be developed in brown, following the same protocol, but without the heat-induced antigen retriever. Alternate sections were counter-stained with Cresyl violet before being dehydrated and mounted with Eukitt (Sigma) mounting medium. Immunofluorescence was performed following the same protocol, but we incubated each primary anti-ACE2 polyclonal antibody (Abcam and Sigma-Aldrich) together with anti-Neuropilin-1 monoclonal antibody during 48 h (RT) in DAKO diluent. Secondary antibodies were Goat anti-Rabbit IgG, Alexa Fluor 488 (green) (Innovative Research Cat# A31565, RRID:AB_1500683; 1/500) Anti-Rabbit IgG, Alexa Fluor 594 nm (red) (Innovative Research Cat# A24923, RRID:AB_1500794; 1/500) Anti-Mouse IgG, and Alexa Fluor 488 nm ( Innovative Research Cat# A31561, RRID: AB_1500651;1/500). After immunofluorescence slides where mounted with Mowiol mounting medium (Sigma-Aldrich Cat# 81,381).

Technical quality controls and immunohistochemical specificity tests were performed in equivalent parallel control sections processed in the same Coplin glass and jars than experimental sections and following the same protocol, but without the primary or secondary antibodies. For each antibody and in all the experiments performed, two slides where incubated in Vision FLEX Antibody Diluent for 48 h (RT), without primary antibodies. In addition, for each antibody and in all the experiments performed, two slides of the series incubated with primary antibodies were incubated in Vision FLEX Antibody Diluent but without secondary antibodies. These negative controls never showed specific staining or significant background (Supplementary Fig. 1). Positive control of ACE2 expression was determined by immunostaining of kidney and lung sections, as well as adult brain sections of our collection banc (Supplementary Figs. 1 and 2), processed in parallel following the same procedure.

### Immunocytochemistry

Cells were plated onto poly-l-lysine (10 μg/ml, Sigma-Aldrich)-coated multi-chambers (BD Falcon) and maintained in basal media. Cells were fixed in freshly prepared 4% paraformaldehyde (PFA; Sigma). Fixed cells were blocked for 1 h in PBS containing 10% normal horse serum (Gibco) and 0.25% Triton X-100 (Sigma) and incubated overnight (O/N) at 4 °C with polyclonal anti-NESTIN (Sigma-Aldrich; C# N5413; RRID:AB_1841032; 1/500) and ACE2 antibodies. Afterward, cells were rinsed and incubated with the corresponding secondary antibodies: Alexa Fluor® 488 (green) (Innovative Research Cat# A31565, RRID:AB_1500683; 1/500) and Alexa Fluor 594 nm (red) (Innovative Research Cat# A24923, and RRID:AB_1500794; 1/500). Cell nuclei were counterstained with DAPI (Molecular Probes, 0.2 mg/ml in PBS).

To quantify ACE2 expression in cultured cells, three random images of the wound area were captured in each condition by a confocal microscope. Prior to quantification, images were de-convoluted by Huygens professional software to improve signal-to-noise ratio (SNR). Sum projections of de-convoluted images were analyzed using Fiji software. Selected cells expressing ACE2 in the wound area (26 cells at t0 and 22 cells at t12) were quantified in terms of fluorescence intensity with the following formula: Corrected Total Cell Fluorescence (CTCF) = Integrated density—(Area of selected cell × Mean Fluorescence of background readings). Non-parametric test for paired (Wilcoxon test) and independent (Mann–Whitney test) samples were used according to Hammond Luke (2014). Measuring cell fluorescence using ImageJ; p.5d853e60. https://theolb.readthedocs.io/en/latest/imaging/measuring-cell-fluorescence-using-imagej.html

## Results

### ACE2 expression in the migratory stream and germinative matrices of the dentate gyrus of hippocampus

**Fig. 1:**
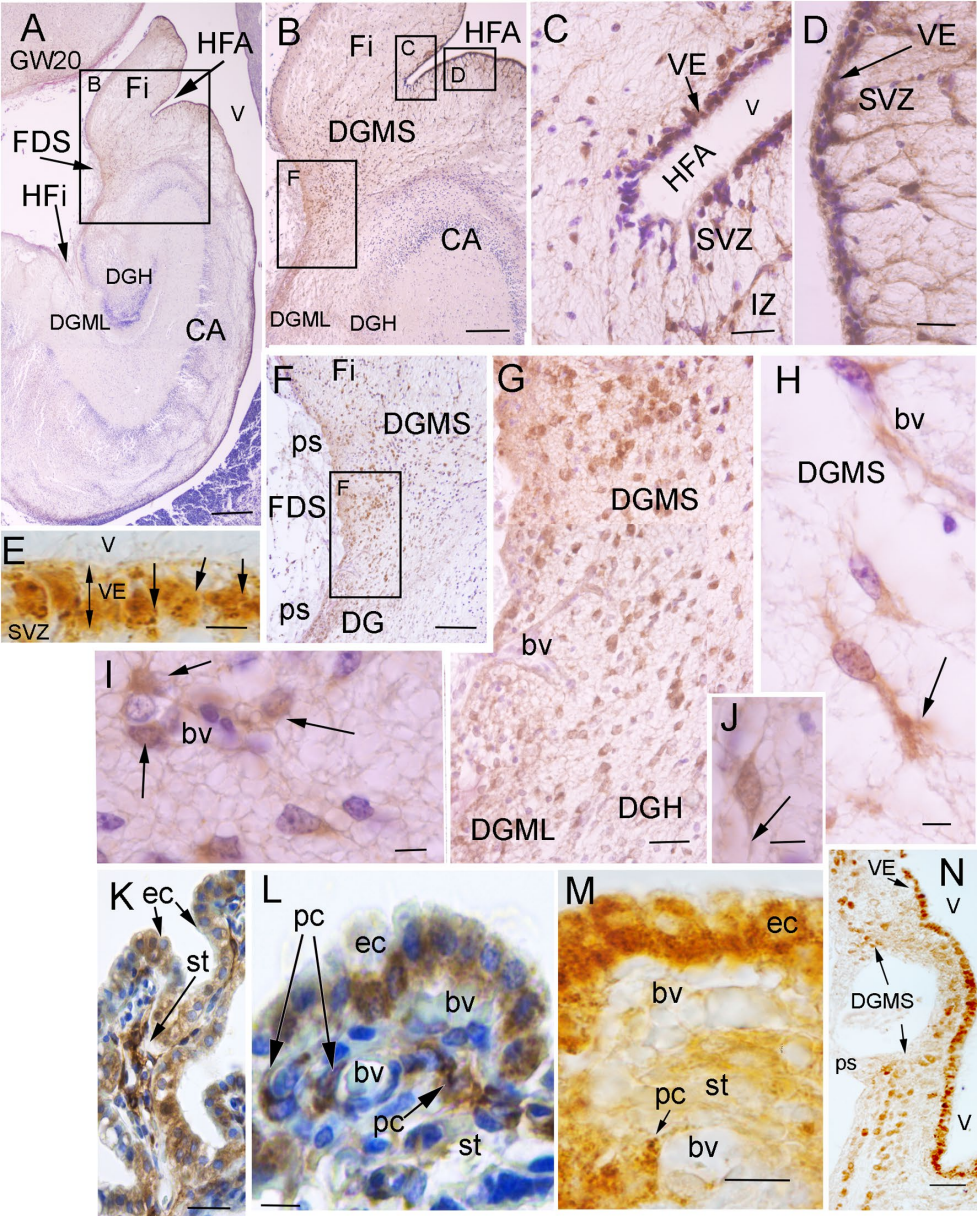
ACE2 expression in human fetal hippocampus. **A** Image of a coronal section of the temporal lobe showing the hippocampal formation. Insert represents the picture showed in (**B**). **B** Picture showing the Hippocampus Fimbrial Angle (HFA) between the fimbria and the Ammon’s horn (CA) ventricular surfaces. Inserts represent the areas that are showed in C, D, and F. ACE2 immunopositive (ACE2 +) migratory cells were observed from the HFA ventricular epithelium (VE) to the subpial region (pial surface: ps), through a radial migratory stream of cells toward the dentate gyrus (DGMS). **C** High power photomicrograph of the HFA showing the distribution of ACE2 + cells in the ventricular epithelium (VE) and the subventricular zone (SVZ). **D** High power photomicrograph of ventricular surface showing ACE2 + cells in VE and the subventricular zone (SVZ) of proximal (**D**) side of the HFA. **E** Ventricular surface showing ACE2 immunopositive particles accumulate on the basal pole membrane of VE progenitors (arrows). **F** Picture showing ACE2 + migrating cells reaching the developing Dentate Gyrus (DG) at the pial surface (ps) of FDS. **G** High power photomicrographs of migratory ACE2 + cells reaching he ps of the DGMS. **H**–**J** High power pictures showing ACE2 expression in the advance processes of migrating cells: (arrows in **J** and **H**), and cells around blood vessels (arrows in **I**). **K**–**M** ACE2 expression in choroidal plexus cells. Both epithelial cells (ec) in the ventricular surface, and stromal pericytes (pc) around blood vessels (bv) were immunopositive (arrows). **N **ACE2 expression in DG ventricular epithelium and DGMS at the caudal pole of hippocampus (FC and IG). Scale bar: **A** 500 µm; **B** 300 µm; **C**, **D** 50 µm; **E** 10 µm; **F** 150 µm; **G**, **N** 100 µm; **H–K** 15 µm; **L**, **M** 5 µm. *bv* blood vessel, *CA* cornu ammonis, *DGMS* dentate gyrus migratory stream, *ec* epithelial cell, *FDS* fimbrio-dentate sulcus, *Fi* fimbria, *HFA* hippocampus fimbrial angle, *pc* pericytes, *ps* pial surface, *SVZ* sub-ventricular zone, *st* choroidal plexus stroma, *VE* ventricular Epithelium

ACE2 immunopositivity was detected in cell populations of the developing hippocampus (Fig. 1). Abundant ACE2 immuno-positive (ACE2 +) cells were observed in the neuroepithelial progenitors (VE) of the dentate gyrus in the hippocampus fimbrial angle (HFA; panels C, D), and the primary germinative matrix of the DG (Fig. 1A–E; [25, 26]); as well as the other hippocampus homologue regions where the DG is represented: fasciola cinerea (FC), indusium griseum (IG; Fig. 1N), and tenia tecta (TT; [27]). From the ventricular progenitors, ACE2 + cells migrated into the subventricular zone (SVZ) and the intermediate zone (IZ), toward the subpial region of fimbrio-dentate sulcus (FDS) under the pial surface (ps; Fig. 1B–K), generating the DG migratory stream (DGMS) between the dentate anlage and the hippocampal plate or Cornu Ammonis [CA], and generated the secondary matrix of DG, according to Altman and Bayer [25, 26]. DG migratory cells expressed ACE2 in the DGMS, from the SVZ to the DG granular layer, with a progressive reduction of ACE2 expression on their pathway to the DG hilux (DGH) and the developing DG molecular layer (DGML; Fig. 1F–J, N). Moreover, additional expression was detected in the ventricular epithelium and SVZ of other brain regions: the epithelial cells and pericytes of the choroidal plexus in the lateral ventricles (Fig. 1K–M) and the epithelial cells of the paraventricular hypothalamus (data not shown). The ACE2 expression data were similar and reproduced in the three brains studied and completely symmetrical in the both sides of each brain. Negative controls of immunostaining without primary or secondary antibodies never showed specific staining or significant background (Supplementary Fig. 1A–C).

**Fig. 2:**
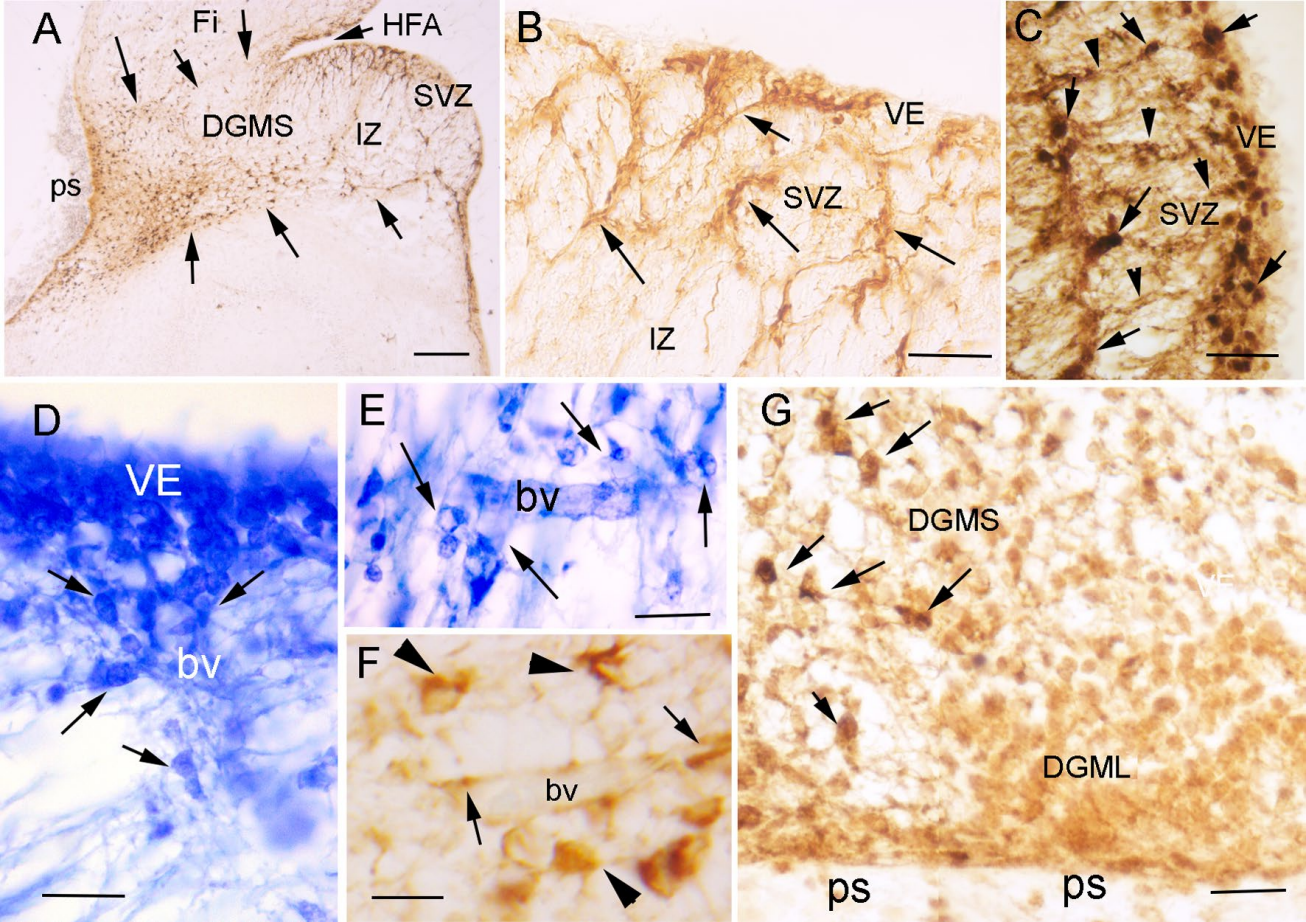
Migration routes of DG progenitors. **A** Picture of a coronal section of the temporal lobe showing GFAP expression in radial glia cells and fibers of the Sub-ventricular Zone (SVZ) and Migratory Stream (DGMS) of the Dentate Gyrus (DG), from the Hippocampus Fimbrial Angle (HFA) to the pial surface (ps). DGMS has been delimited by arrows. **B** High power picture of the DG Ventricular Epithelium (VE), Sub-ventricular Zone (SVZ) and intermediate zone (IZ). Arrows label GFAP positive fibers. **C** High power picture of ACE2-expressing cells (cells with DAB-nickel precipitate; arrows) following GFAP radial glia fibers of DG ventricular epithelium (VE; DAB precipitate; arrows head). **D**, **E** Cresyl violet staining showing the DG ventricular (VE) and sub-ventricular (SVZ) zones with perivascular accumulation of cells crossing the sub-ventricular withe matter (alveus) in the DG migratory stream (DGMS; arrows). **F** ACE2 expression in perivascular migrating cells (arrowheads) and pericytes (arrows) in the alveus. **G** Doublecortin expression in DG migrating neuronal precursors and neurons in DG molecular layer (DGML). ACE2 is expressed in migratory precursors of the DGMS (DAB-nickel precipitate; arrows) but not in DGML, single doublecortin expressing cells (DAB precipitate). Scale bar: **A** 200 µm; **B** 100 µm; **C**–**E** 50 µm; **F** 25 µm. *bv* blood vessel, *DGH* Dentate Gyrus Hilux, *DGMS* Dentate Gyrus Migratory Stream, *DGML* Dentate Gyrus Molecular Layer, *HFA* Hippocampus Fimbrial Angle, *ps* pial surface, *VE* ventricular epithelium

The ACE2 immuno-precipitate presented a granular pattern in the cytoplasm of ventricular layer progenitors (arrows in Fig. 1C–E) and migratory cells along the DGMS, with more staining accumulation in the exploration cones of advance processes (arrow in Fig. 1H). Immunopositivity decreased in the migratory cells following a superficial stream and entering the DGML and in a deep migratory stream to the DGH (Fig. 1F, G). Once the ACE2-expressing cells reached the SVZ and entered the DGMS, they followed the direction of radial glia fibers that were detected by GFAP expression (Fig. 2A–C). In the SVZ of the CA and HFA, the migrating cells appeared as radial chains of cells (arrows in Fig. 2B, C). They also accumulated around the blood vessels that orthogonally cross the axonal fibers in the alveus (arrows in Fig. 2D), suggesting that perivascular spaces represent preferential pathways for radial glia fiber accumulation and cellular migration across the alveus, which is formed by pyramidal cell projections of CA to the fimbria. At more superficial levels, inside the DGMS, perivascular migratory cells were frequently detected (Fig. 2E, F). At the brain surface, next to the pial surface (ps) of FDS region of the DGMS, the cells differentiated into migratory neurons expressing doublecortin and the expression of ACE2 progressively decreasing (Fig. 2G).

To confirm that the observed expression pattern of ACE2 is specific, we have performed immunolabeling experiments with two other antibodies, as well as positive expression controls in human kidney and lung sections for each of them, as well as adult hippocampus. In addition to the rabbit polyclonal anti-ACE2 from Abcam, we have processed parallel sections using another rabbit polyclonal anti-ACE2 from Sigma-Aldrich and a mouse monoclonal anti-ACE2 from R&D Systems. We have confirmed the sensitivity of these antibodies by detecting specific expression in lung alveolar and renal proximal tubule cells (Supplementary Fig. 2A–F), as has been reported in previous studies [18, 28]. The immunostaining observed in the parallel processed sections was similar and specific in VE dentate gyrus progenitors and migrating cells in DGMS (Supplementary Fig. 2I–J). Parallel series of control sections processed without primary antibodies did not show any specific immunostaining or significative background, and were counterstained with Cresyl violet (Supplementary Fig. 2G, H).

### ACE2-expressing cells in the hippocampus are migratory neural progenitors

**Fig. 3:**
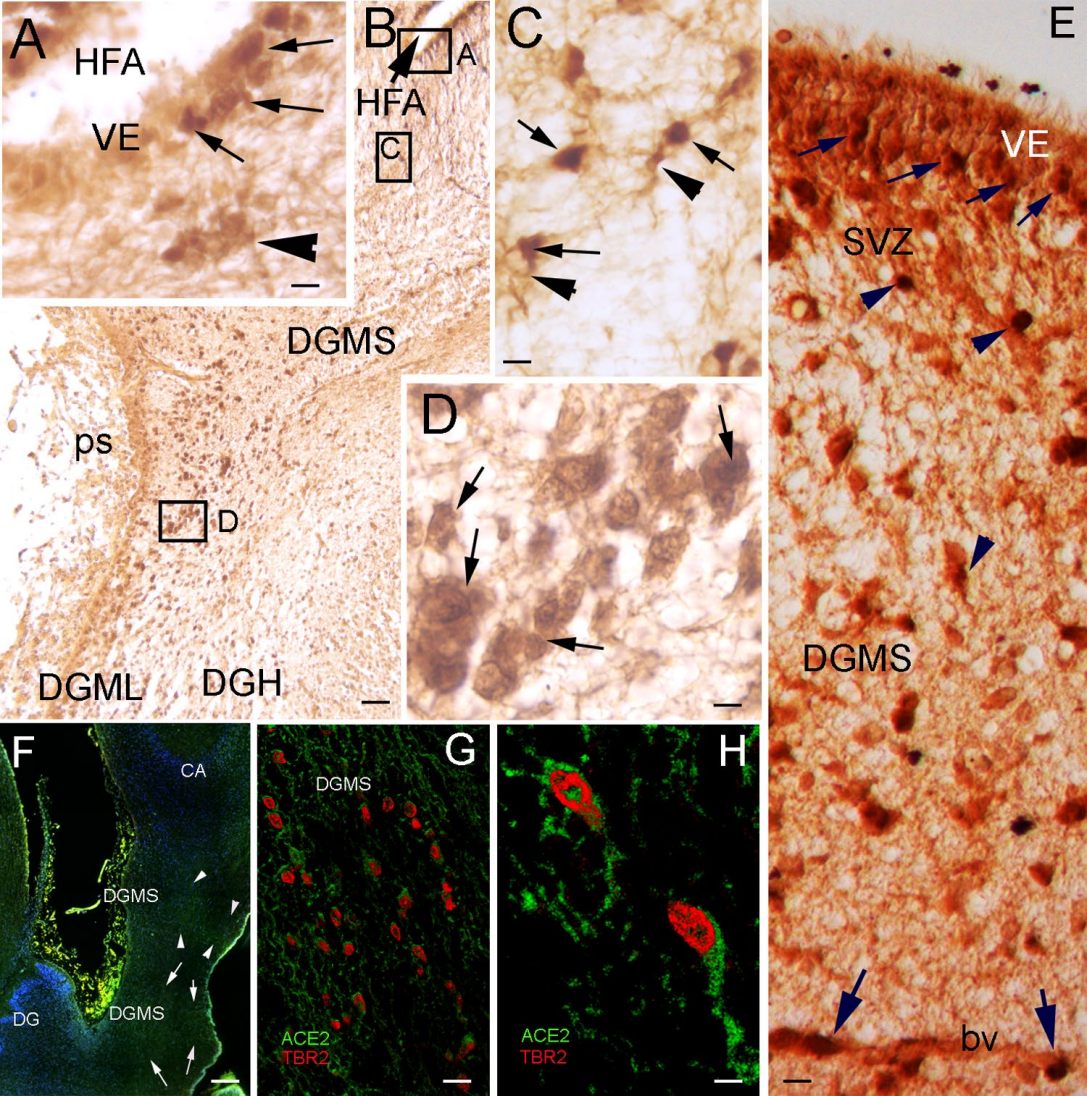
ACE2 + cells in DGMS are TBR2 + neural progenitors. **A** High power picture showing DG ventricular epithelium (VE) expressing ACE2 and TBR2 in the Hippocampal Fimbrial Angle (HFA). Double immunopositive, ACE2 and TBR2, cells are mainly localized in the basal surface of the VE (arrows) and in cells that are migrating into the subventricular zone (arrowhead). **B** Microphotograph showing the Dentate Gyrus Migratory Stream (DGMS) from the HFA to the pial surface (ps), where DGMS migratory cells follow dorsally to the Dentate Gyrus Hilux (DGH) or ventrally to Dentate Gyrus Molecular Layer (DGML). Insets localized the areas showed in (**A**, **C**, and **D**). **C** High power microphotograph showing ACE2 (DAB precipitate in the cytoplasm; arrowheads) and TBR2 (DAB-nickel precipitate in the nucleus; arrows) expression in ventricular and migratory cells. **D** High power of the zone represented in the inset of **B** showing double labeled ACE2 and TBR immunopositive cells in the DGMS (arrows). **E** Picture showing DG ventricular epithelium (VE) expressing ACE2 and TBR2 at the Hippocampal Fimbrial Angle (HFA) (small arrows) and in migrating cells in the SVZ and DGMS (arrowheads). Large arrows show ACE2 and TBR immunopositive cells migrating near to a blood vessel (bv). **F** Low power picture of an immunofluorescence processed section, showing the DGMS at the caudal pole of the hippocampus, where the DG turns dorsally to became the fasciola cinerea. Ventral and dorsal DGMS are delimited by arrows. **G**, **H** High power picture of immunofluorescence showing ACE2 immunolabeling in the cytoplasm (green) and TBR2 immunolabeling in the nucleus (reed) in DGMS cells. Scale bar: **A**, **C**, **E**, **G** 10 µm: **B** 200 µm; **D**, **H** 7 µm; **F** 300 µm. *bv* blood vessel, *CA* Ammon’s horn, *DGH* Dentate Gyrus Hilux, *DGML* Dentate Gyrus Molecular Layer, *DGMS* Dentate Gyrus Migratory Stream, *HFA* Hippocampus Fimbrial Angle, *ps* pial surface, *SVZ* sub-ventricular Zone, *VE* ventricular epithelium

To demonstrate whether ACE2 + cells are DG progenitors, we performed double immunohistochemistry, showing that ACE2 is expressed in TBR2 immunopositive (TBR2 +) cells of the DG ventricular neuroepithelium and DGMS (Fig. 3), which are intermediate migratory progenitors in the hilux and molecular layer of the DG, at the FDS ps surface (Fig. 3A–E). Once the progenitors reached the DGML, they stopped expressing ACE2 (Fig. 3B). Double immunofluorescent experiments showed the colocalization of ACE2 and TBR2 in the cytoplasm and nucleus, respectively, of migrating cells in the DGMS (Fig. 3F–H). Double immunohistochemistry therefore showed that the ACE2-expressing cells are the TBR2 + , a specific marker for this population, and they colocalize with GFAP immunopositive cells in the same brain areas: the SVZ, IZ and DGMS. In Cipriani et al. [29] and in human samples from the Allen Brain Institute database, DG progenitors specifically express *TBR2* gene along the migratory pathway, from the ventricular epithelium and SVZ to the pial surface of the FDS.

Migration of DG precursors in the DGMS followed radial glia fibers [30] and (Fig. 2A–C). Hence, cells along this radial migration expressed ACE2 throughout the migratory pathway from the SVZ to the DG hilux (DGH) and pial surface. Therefore, ACE2 + /Tbr2 + precursors migrated radially along the DGMS to the superficial region (Fig. 3A–C). From this area, they followed a deep migration route to the DG hilux, and a superficial migration route toward the DG molecular layer. ACE2 expression progressively disappeared and cells disseminated into the DG molecular and granular layers (Fig. 3B).

### ACE2-expressing cells in the hippocampus express Neuropilin-1 receptor

**Fig. 4:**
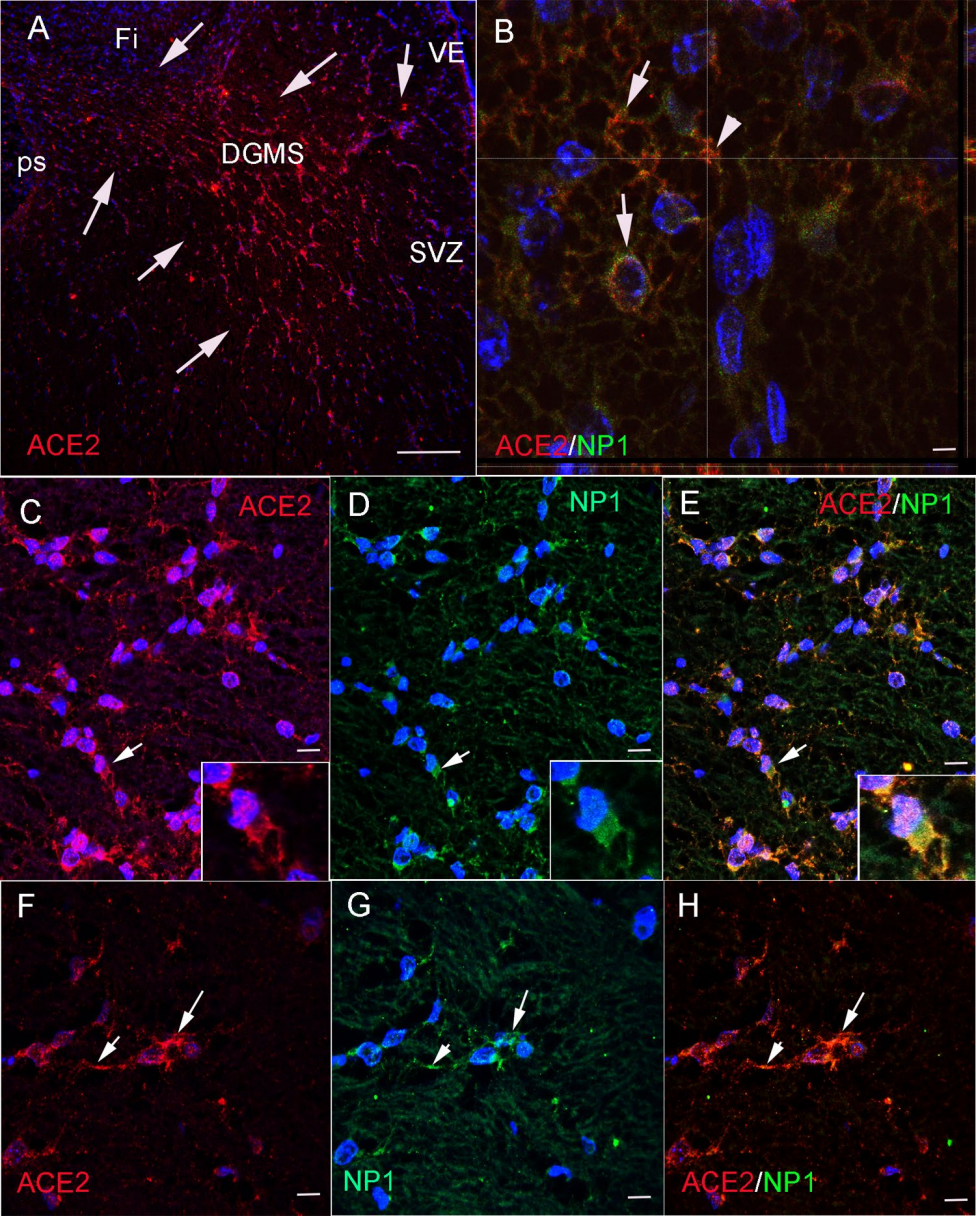
ACE2 and neuropilin 1 (NP1) co-localize in the membrane of DGMS cells. **A** ACE2 immunofluorescent cells in the DGMS between sub-ventricular zone of the HFA and the ps (arrows). **B** High power confocal photomicrograph sowing the co-expression of ACE2 and NP1 in the membrane of migrating cells (arrows). The arrowhead shows the point where Z-stack projection have been reconstructed (view in the x and z plane), to demonstrate co-localization of ACE2 (reed dots) and NP1 (green/yellow dots) in the cellular membrane. **C**–**E** Microphotographs showing ACE2 and NP1 co-expression in DGMS cells. Inserts show the same cells with clear co-expression of ACE2 and NP1 in the membrane of leading processes (arrows). **F**–**H** DGMS cell showing strong co-expression of ACE2 and NP1 in the cellular membrane of leading processes (arrows). Scale bar: **A**: 250 µm; **B** 10 µ; **C**–**E** 20 µm; **F**–**H** 15 µm

Neuropilin-1 (NP1) expression at the cell membrane of olfactory epithelium cells has been shown to facilitate SARS-CoV-2 cell entry and infectivity [31]. Since NP1 is expressed in hippocampal migratory neurons [32], we have explored co-expression of ACE2 and NP1 in the developing human hippocampus in order to reinforce our data showing that migratory newborn neurons are a privileged target of SARS-CoV2 infection in the developing brain (Fig. 4). By immunofluorescent experiments, our human fetal sections showed strong expression of ACE2 in the DG ventricular epithelium, SVZ and DGMS, the same areas which have been mapped in DAB processed sections (Fig. 4A). Double immunofluorescent methods using ACE2 and NP1 antibodies revealed co-localization of these two receptors in migrating cells of DGMS (Fig. 4B–H). Therefore, ACE2 and NP1 are co-expressed in neural cells, in a similar way as reported in olfactory epithelium cells [32], where NP1 expression in cells of DG may potentiate SARS-CoV-2 infectivity.

### Cell migration and ACE2 expression

**Fig. 5:**
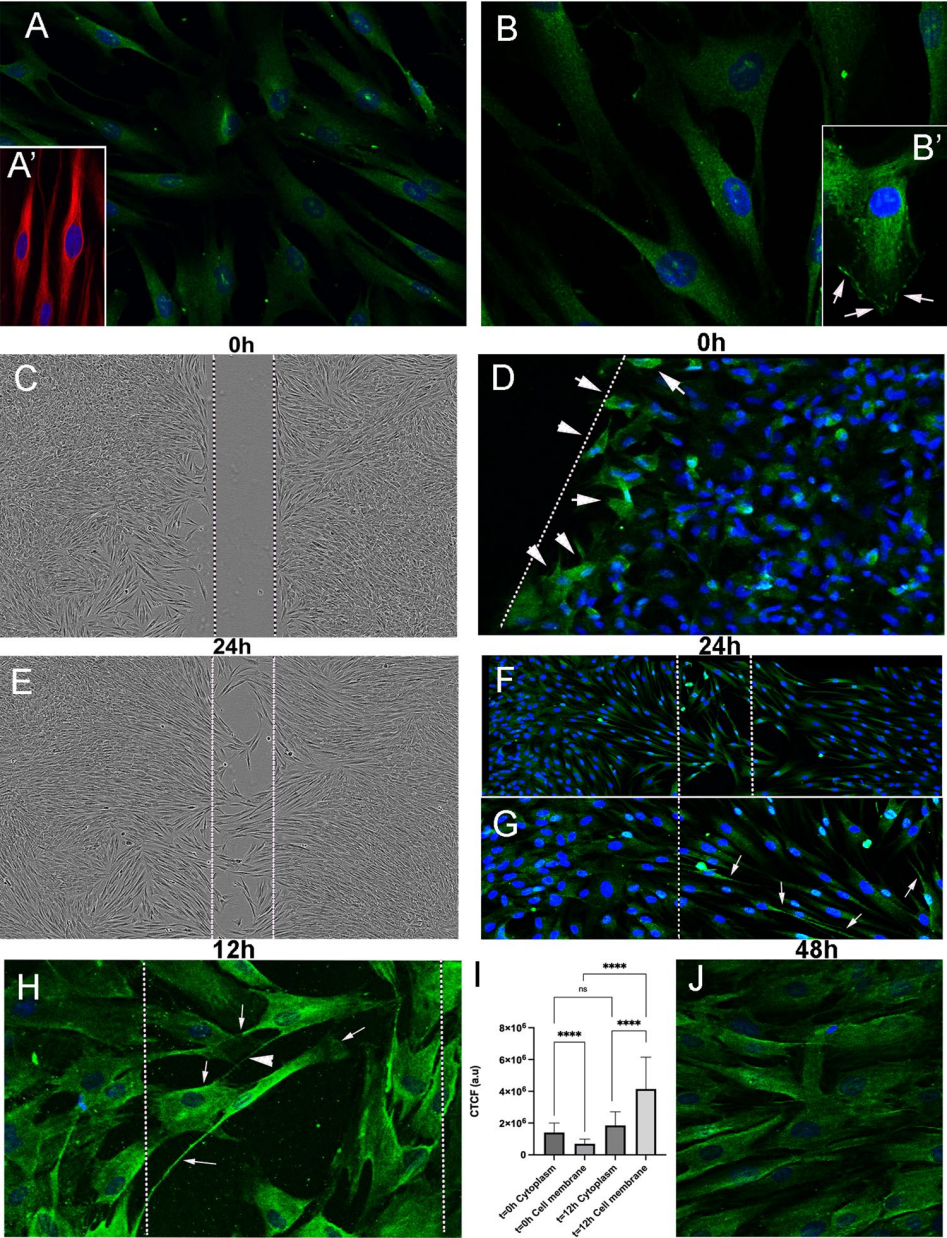
ACE2 expression in migrating neural crest progenitors. **A**, **B** Anti-ACE2 immunofluorescence showed expression of ACE2 in the cytoplasm of cultured dental derived NCP, with perinuclear accumulation. **A’** Dental derived NCP express Nestin protein (red immunofluorescence). **B’** Cells with polarized morphology ACE2 expression is stronger and accumulated in the cellular membrane of the progression pole (arrows). **C**–**I** Scratch-wound assay in confluent cultures. **D** 10 min after the scratch (t0h) cells at the wound edge strongly expressed ACE2 (arrows). **E**–**I** At 12–24 h (t12h, t24h) after the scratch, polarized elongated cells migrated into the wound with strong expression of ACE2 in the cellular membrane of advance processes (arrows) including in intercellular nanotubes (arrows head in **H**). **I** Quantification of ACE2 expression by immunofluorescence intensity and intracellular distribution with the ImageJ tool for measuring corrected total cell fluorescence (CTCF). Data shown as mean ± S.D. The number of cells analyzed were: T0h (cytoplasm or membrane) *n* = 26, T12h (cytosol or membrane) *n* = 22. Comparisons between cytoplasm and cell membrane at T0h and at T12h were made with tests for paired samples (Wilcoxon signed-rank test) and comparisons between cytoplasm at T0h and T12h or cell membrane at T0h and T12h were made with test for independent samples (Mann–Whitney test for T0h test), using the software GraphPad Prism. *a.u* arbitrary units. Asterisks indicate *p*-value: **** *p* < 0.0001 *ns* not significant. **J** At 48 h after the scratch the wound was completely cellularized with increased expression of ACE2 in some cell membranes of neighboring cells at the place where the wound was performed (arrows)

**Fig. 6:**
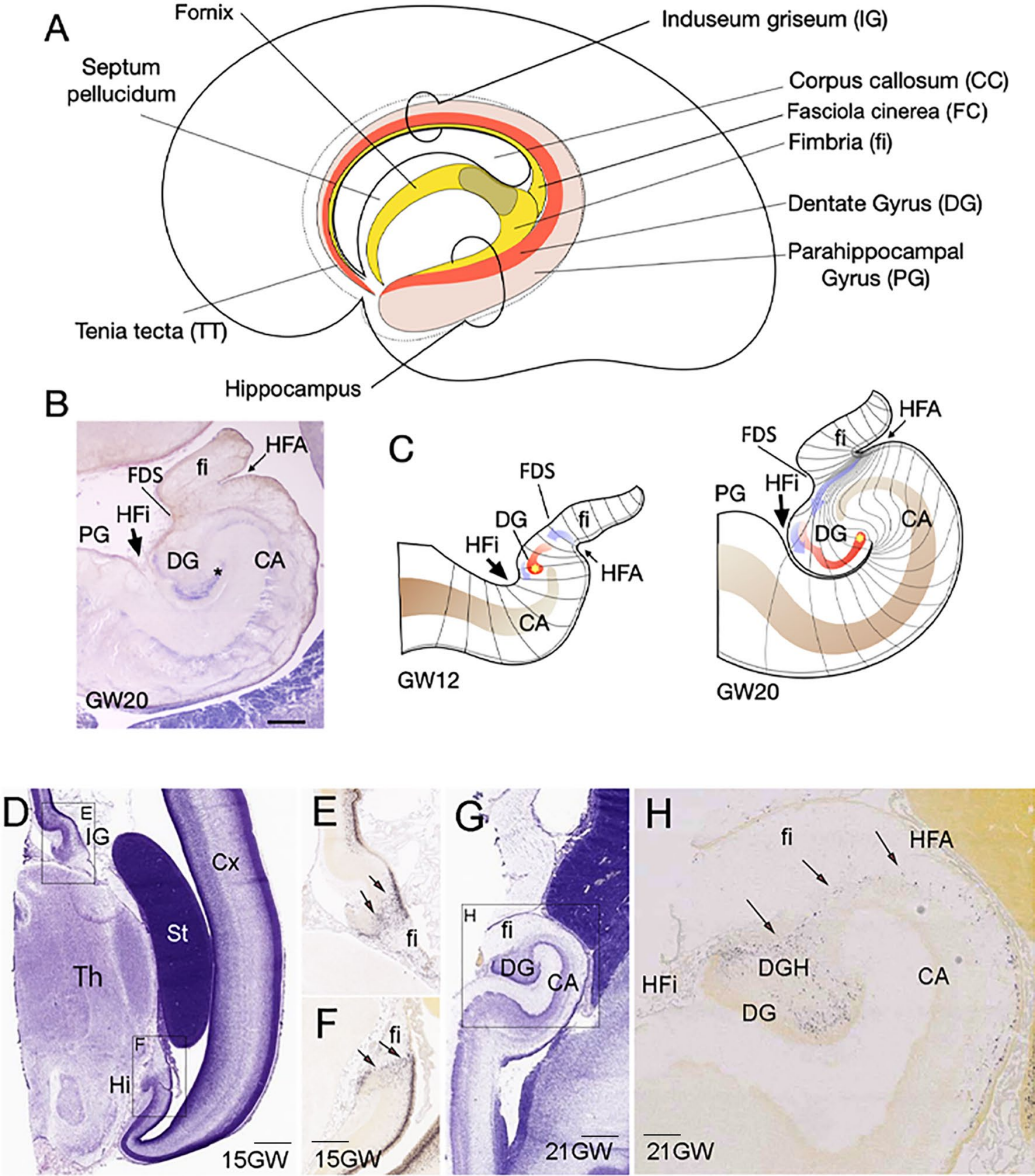
Dentate gyrus morphogenesis. **A** Schematic representation of a medial view of the brain left hemisphere of the 20 gestational weeks (20GW) human fetus, where hippocampal structures have been identified. In the retro-commissural and temporal regions of the medial pallium (posterior and ventral to the corpus callosum, CC) the hippocampus is formed by the fimbria (fi), the most medial structure; the dentate gyrus (DG) develops in the distal pole of the medial pallium (hippocampal formation) and it is concentric to the hippocampal cortex (Cornu Ammonis: CA). In the anterior regions of the medial pallium: the indusium griseum (IG), the fasciola cinerea (FC) and the tenia tecta (TT) represent, respectively, the supracommissural and precommissural DG. **B** Transversal section of hippocampal formation from a 20 weeks human fetus. The section has been stained with Cresyl violet to identify the cellular distribution: parallel to the ventricular surface, the developing cortex of CA folds toward the pial surface at its medial pole and continues with the DG hilux. The DG developing cortex is formed with a medio-lateral gradient of cellular layer maturation (the lateral region [asterisk] is more mature that medial region). The hippocampal fissure (HFi) is visible at the pial surface between DG and para-hippocampal gyrus (PG). The ventricular surface shows the hippocampus fimbrial angle (HFA) between the CA and fi. (Scale bar: 500 µm). **C** Representation of medial pallium (MP) deformation in the process of DG morphogenesis. The radial axis deformation is showed at different regions by lines to represent the compaction of radial glia axis at the medial pole of DG due to the folding of DG anlage. DG is represented in red and more mature lateral pole is labeled by an asterisk. Blue arrows indicate the migration of DG progenitors. **D** Cresyl violet staining of a human brain at 15GW. The hippocampus (Hi) and indusium griseum IG) regions were localized at the ventral and dorsal regions of medial pallium (insets show the regions in **E** and **F**). **E**, **F** High power pictures of CA and DG where in situ hybridization (ISH) detected *TBR2* gene expression (images from Developing Human Brain project of Allen Institute (https://www.brainspan.org/rnaseq/search/index.html, were captured directly from the ISH-Atlas). The migratory *TBR2* + cells in the Dentate Gyrus Migratory Stream (DGMS) were labeled by arrows. **G** Cresyl violet staining of a human brain at 21GW. **H** High power of coronal sections of human hippocampus at 21GW. In situ hybridization (ISH) detecting *TBR2* gene expression was illustrate. The DGMS was labeled by arrows, showing *TBR2*-expressing cells from the HFA to Hippocampal Fissure (HFi). Scale bar: **A** 500 µm; **D**, **G** 300 µm; **E**, **H** 200 µm. *CA* cornu ammonis, *DG* dentate gyrus, *fi* fimbria, *FDS* fimbrio-dentate sulcus, *HFA* hippocampus fimbrial angle, *HF* hippocampus fissure, *Hi* hippocampus, *IG* indusium griseum, *Mes* mesencephalon, *PN* pontine nuclei, *St* striatum, *Th* thalamus

To explore the possible regulation ACE2 expression in other model of human migratory cells, we have selected neural crest progenitors (NCP) in vitro. Neural crest cells are a transient cell population in the embryo that exhibits a variety of migratory mechanisms, including sheet and chain migration, in which NP1 expression is required [reviewed by [33]. Since dental pulp and ligament cells derive from NCP, human teeth may represent a source of human neural crest cells. Cells from dental premolars were cultured following the protocol described in material methods according to Bueno et al. [34] to derived mesenchymal stem cells that were molecular and functionally characterized as NCP [34]. The expression of Nestin has been used to confirm that our cultured cells are NCP (Fig. 5A, A’). ACE2 immunofluorescence showed expression of ACE2 in the cytoplasm of cultured dental derived NCP, with perinuclear accumulation (Fig. 5A, B). In cells with polarized morphology, which were suggestive of cell motility, ACE2 expression was stronger than in basal static fusiform or polygonal cells and also accumulated in the cellular membrane of the leading pole (Fig. 6B’). To explore if ACE2 expression is modified by migratory activity, we have used scratch-wound assay. Cells were grown to confluence and a thin "wound" introduced by scratching with a pipette tip. Cells at the wound edge polarized and migrated into the wound space. Then minutes after the scratch (t0h), cells were fixed and processed by immunofluorescence to detect ACE2 expression. The cells at the wound edge strongly expressed ACE2 (Fig. 5C, D). At 12–24 h (t12h, t24h) after the scratch, polarized elongated cells migrated into the wound with strong expression of ACE2 in the cellular membrane of leading processes and in intercellular nanotubes (Fig. 5E–I). At 48 h after the scratch, the wound was completely cellularized, with remnant expression of ACE2 in the cell membrane of some cells at the place where the wound was performed (Fig. 5 J). After quantification of ACE2 expression at 0 and 12 h after scratch, the increasing expression of ACE2 in migrating cells was significative in the cell membrane during a period of 12 h (the time with active cellular migration into the wound) without modification of ACE signal in the cytoplasm (Fig. 5I).

## Discussion

The COVID-19 pandemic has progressed through several waves of infection as a consequence of different viral streams originated by viral genome mutations. Indeed, the most important viral variants described so far, delta and omicron, improved infectiveness among humans by the increasing the binding affinity of the SARS-CoV-2 trimeric spike glycoprotein to the ACE2 receptor in host cells [35]. Although the severity of COVID-19 has decreased in the most recent waves of the pandemic, due to natural immunization and vaccination, the exposure of the global population to SARS-CoV-2 has been progressively increasing through time. In patients suffering moderate or severe COVID-19, strong evidence for neural effects due to the viral neurotropism of SARS-CoV-2 [13, 14], and/or virus-induced neuroinflammation has been reported [36, 37]). Actually, neurological and cognitive deficits have been demonstrated in COVID-19 patients [38–40], with an incidence of neurological symptoms in more than 80% of severe cases [41]. Postmortem tissue analyses have demonstrated effects on the brains of COVID-19 patients [42, 43], as well as the presence of the coronavirus in the central nervous system [1, 18, 22]. In a longitudinal study, Douaud et al. [44] reported gray matter reduction in limbic structures related to the olfactory system, which may be the consequence of a primary viral effect due to direct viral infection and/or secondary retrograde neurodegeneration from primary infected neurons in the olfactory tubercle and nucleus. Fetal brains may therefore be infected via materno-fetal transmission due to the viral permeability of the immature BBB and/or through peripheral nerves (olfactory and/or pharyngeal nervous plexus), which may trigger neuronal alterations at functional or structural levels, thus generating neurodevelopmental anomalies with the potential to induce neural vulnerability throughout life.

Although materno-fetal transmission of SARS-Cov-2 has not been a frequently reported phenomenon during the COVID-19 pandemic, the large proportion of the human population that has and will be infected, together with the progressively increasing numbers of young people infected at a fertile stage, make it urgent the analysis of the potential consequences of fetal infection by the virus. In a recent review by Piekos et al. [12], the effect of maternal SARS-CoV-2 infection on birth outcomes was studied and concluded that negative birth outcomes were frequent in COVID-19 patients and unrelated to the severity of the disease. Indeed, they conclude that SARS-CoV-2 infection in early pregnancy is a risk factor that needs to be monitored until delivery and even postnatally. Although indirect neural effects secondary to endothelial and neural inflammation may be a pathogenetic process after vascular dissemination of the virus, primary alteration due to neural cell infection is also a possible source of neurological consequences from SARS-CoV-2 infection [13, 14]. For this reason, it seems important to determine which fetal cells and tissues are susceptible to infection, in order to assess the long-term impact of COVID-19 throughout an individual’s life as a sequela of infection during pregnancy. Nevertheless, at the present, it is still too early to observe any long-term impact of in utero SARS-CoV-2 exposure during development. However, mental performance, intellectual development, and incidence of mental diseases should be monitored as it is well known that viral infection during pregnancy increases the subsequent risk of developing neuropsychiatric diseases [45].

Precursors of DG are generated in the most dorsal region of the pallium, the medial pallium, during brain development. This region has, from anterior to posterior, in three regions around the corpus callosum commissure: the precommissural tenia tecta (TT), the supracommissural indusium griseum (IG), as well as the retrocommissural fasciola cinerea (FC), and the hippocampal complex (Hi) [46, 47]. While TT, IG, and FC remain vestigial structures, the hippocampus represents the most developed and complex structure (Fig. 6A). The hippocampus is the brain area that contains the neurons and circuits necessary for acquiring new memories. For instance, hippocampal complex comprises two cortical regions, the Cornu Ammonis (CA1–CA3) and dentate gyrus (DG), the latter of which continues medially to the fimbria (fi; Fig. 6 A, B). During development, between gestational weeks 12 and 22 (GW 12–22), the medial bending of the DG primordium forms the hippocampal fissure (HFi) at the pial surface, and drives DG morphogenesis, with the formation of the hippocampus fimbrial angle or recces (HFA) at the ventricular surface. This morphogenetic movement determines the compaction of radial glia fibers from the HFA germinative epithelium to the HFi, in a corridor with the radial fibers deforming as an open fan toward the DG (Figs. 2A, 6C). Thus, DGMS cells follow a strict radial migration from the HFA to the DG co-expressing NG2/TBR2 (Fig. 3) and NG2/NP1 (Fig. 4). While proximal radial fibers from CA side of the HFA, drive DG progenitors into the hilux, distal radial fibers from fi side of HFA, drive DG progenitors into the molecular and granular layers (Fig. 6C). Between GW15 and 25, hippocampal progenitors are generated and migrated into the developing medial pallium [48]. We have shown that at GW20, the migration of DG progenitors in the hippocampus was identified through the expression of the TBR2 (Fig. 3). To confirm TBR2 expression in DGMS, we have studied *TBR2* expression in the Developing Human Brain project a transcriptomic database using in situ* hybridization* from the Allen Institute (https://www.brainspan.org/rnaseq/search/index.html). In agreement to our results, *TBR2*-expressing cells were detected in the HFA and DGMS (arrows in Fig. 6D–H), the primary and secondary matrix of the DG, described by Altman and Bayer and Cipriani et al. in rodents [25, 26, 29] and Zhong et al. in humans [41].

Sensorial information (visual, auditory, somatosensory, olfactory, and vestibular) for creating spatial and temporal memories is processed in a circuit from the entorhinal cortex (EC) projecting into the DG, then, from the DG to CA3 pyramidal cells, and from CA3 to CA1 pyramidal cells, finally being stored in other cortical circuits in diverse cortical regions. Each one of these hippocampal regions has specific cell types involved in this tri-synaptic circuit [reviewed by 49, 50]. Therefore, viral infection of DG neuronal progenitors during DGMS development may interfere in the proliferation, migration and differentiation of these cells, which could produce a structural alteration and vulnerability to developing cognitive problems during life. Alterations in the DG cell distribution have been described as underlying temporal lobe epilepsy and associated comorbidities, such as memory disturbances and cognitive dysfunction [51]. It seems important to promote longitudinal studies of children exposed to SARS-CoV-2 during their development, as the DG is a cortical region of the limbic system that plays an essential role in memory consolidation, spatial information processing and navigation [52, 53].

An interesting issue is the physiological role of ACE2 expression in migratory cells and hippocampal progenitors. Processing of Ang II by ACE2 enzyme renders Ang-(1–7), a metabolite that activates the mitochondrial assembly (Mas) receptor (MasR) pathway and increases the vascular supply to ACE2-expressing cells [54]. Moreover, MasR activation has beneficial effects, facilitating vasodilatation and increasing anti-inflammatory and antioxidative responses [17]. In addition, Ang II receptors are highly expressed in developing neurons and have been associated with neurite growth and neural cell migration [16, 55], maintaining the undifferentiated stage of neural progenitors [56], and proliferation in the DG [57]. ACE2 expression in DG migratory progenitors may therefore be a key factor in decreasing Ang II vasomotor activity in the ventricular epithelium, as well as the SVZ and along the DGMS. These progenitors accumulate around the blood vessels in the SVZ of DG to traverse the alveus and migrate into the DGMS. Since there is no significant synthesis of angiotensin in the brain, the vascular system is the source of Ang II [58]. This implies cellular tropism to the vascular system, may represent a mechanism for improving vasodilatation and oxygen supply, as well as neurotrophic activity in migratory progenitors that express ACE2. Our results in neural crests progenitors in vitro suggest that increasing ACE2 expression and mobilization to the cell membrane may represent a general mechanism present in migratory cells to improve oxygen supply along migration pathways.

Our results are a descriptive analysis of ACE2 expression in the human fetal brain at a cellular level. We are aware that the study of only 3 brains might be an important weakness of this descriptive work, but the complexity of obtaining good-quality fetal human brains that have been expertly dissected, fixed, and histochemical processed means that this is a very rare opportunity for studying the potential effects of COVID-19 on the human fetal brain. The ACE2 expression maps were very similar in the three brains and on both sides of each brain, demonstrating the reproducibility and increasing the plausibility of these maps. Moreover, positive sensitivity and specificity of immunostaining have been proved by detection of expression in lungs and kidneys, corroborating the reported expression of ACE2 in adult human tissues [28]. In addition, we have described the presence of the caudal migratory stream expressing TBR2 along the GFAP + radial glia fibers, from the fimbria hippocampus angle to the marginal layer of the DG, which generates intermediate DG progenitors during the first two postnatal weeks of brain development [59], and corroborated the data published by Ciprinai et al. [29]. Moreover, we have also demonstrated the close interaction between blood vessel development and migratory neural progenitors in mouse DG development at these early postnatal stages [60]. The developmental timing of the human hippocampus at 16–20 gestational weeks [48] is similar to that of mice at postnatal 0–7 days, the stage at which we have described this migration. Therefore, the migratory stream described in DG development in mice is present in the human brain at 20 gestational weeks. Finally, we have also detected ACE2 expression in adult hippocampus (dentate gyrus) astrocytes and pericytes, in agreement with Chen et al. [61], which suggest the potential infectivity of adult neural cells.

In conclusion, neurological COVID-19 disorders constitute a major public health challenge not only due to the acute and long-term effects on the brain [40, 41], but also the congenital infection and neurodevelopmental disorders that may ensue. For this reason, clinical and laboratory efforts aimed at elucidating the mechanisms of the acute effects on the brain of SARS-CoV-2 need to be coupled with research into any deleterious delayed neuropsychiatric sequelae of the infection in children affected by the vertical transmission of the virus. Close cooperation between clinical and basic scientists is required to tackle these potential effects and take advantage of the wealth of clinical–epidemiological data and biological specimens that are accumulating around the world. Considering that the COVID-19 pandemic is still raging in many countries, there may be a seasonal resurgence of vertical viral transmission, and it is imperative that a concerted effort is implemented swiftly and on a large scale.

## Supplementary Information

Below is the link to the electronic supplementary material.

Supplementary Figure 1: Control of immunolabelling and ACE2 expression in adult hippocampus. A-C) Pictures of the hippocampus fimbrial angle in control processed sections without primary ant-ACE2 antibodies: A) rabbit polyclonal anti-ACE2 (Abcam Cat# ab15348). Control sections processed with rabbit polyclonal anti-ACE2 (Sigma-Aldrich Cat#HPA000288) and mouse monoclonal anti-ACE2 (R&D Systems Cat# MAB933) were similar to this one (data not shown). B) rat monoclonal anti-GFAP (Millipore Cat# 345860-100UG). Control sections processed with goat polyclonal anti-Doublecortin (Santa Cruz Biotechnology Cat# sc-8066) were similar to this one (data not shown); C) rabbit polyclonal anti-TBR2 (Abcam Cat# ab23345). D-G) ACE2 expression has been detected in paraffine section of adult hippocampus, demonstrating specific expression of ACE2 (rabbit ati-ACE2 from Abcam) in astroglia in D, E and F (arrows), and pericytes in G (arrow). While ACE2-expressing cells are identified by brown-colored neurons were negative as we can observe in D by the white color (arrowhead). H) Double immunohistochemistry showing GFAP expression (brown) and ACE2 expression (black dots; arrows) in astroglial cells. I) Control section processed with GFAP and without ACE2 antibodies, showing the GFAP derived immunoreactivity and absence of ACE2 immunostaining. Scale bar: D-I) 50 µm

Supplementary Figure 2 is not the revised version and has to be changed by the uploaded one, which contains the revised lettering: Specificity of ACE2 expression in human lung, kidney and developing brain. A-C) The three antibodies show ACE2-specific immunoreaction in human lung in alveolar endothelial type II cells (arrows) and capillary endothelium (arrowheads). D-F) The three antibodies show ACE2-specific immunoreaction in human renal proximal tubule cells (arrows). G) Low power picture of a brain section processed in parallel by immunohistochemistry without primary ACE2 Sigma-Aldrich antibody and counterstained with Cresyl violet. H) High power picture of a brain section processed in parallel by immunohistochemistry without primary ACE2 R&D antibody and counterstained with Cresyl violet. Control sections without primary antibodies do not showed immunostaining. I-N) The three antibodies show ACE2-specific immunoreaction in DG progenitors and migrating cells in DGMS. Scale bar: A, B, C) 50 µm; D, E, F) 25 µm; G, I, K, M) 100 µm; J, L, N) 25 µm. DGMS: Dentate Gyrus Migratory Stream; IZ: Intermediate Zone; SVZ: Subventricular zone: VE: Ventricular Epithelium

## Data Availability

Not applicable.
