# Supplementary figures and images for: Neuronal progenitors of the dentate gyrus express the SARS-CoV-2 cell receptor during migration in the developing human hippocampus
Source: Cell Mol Life Sci. 2023 May 7;80(6):140. doi: 10.1007/s00018-023-04787-8 (PMC10164240; doi:10.1007/s00018-023-04787-8)

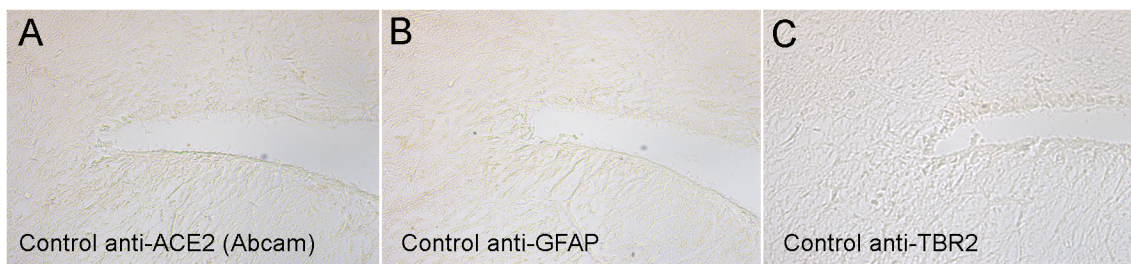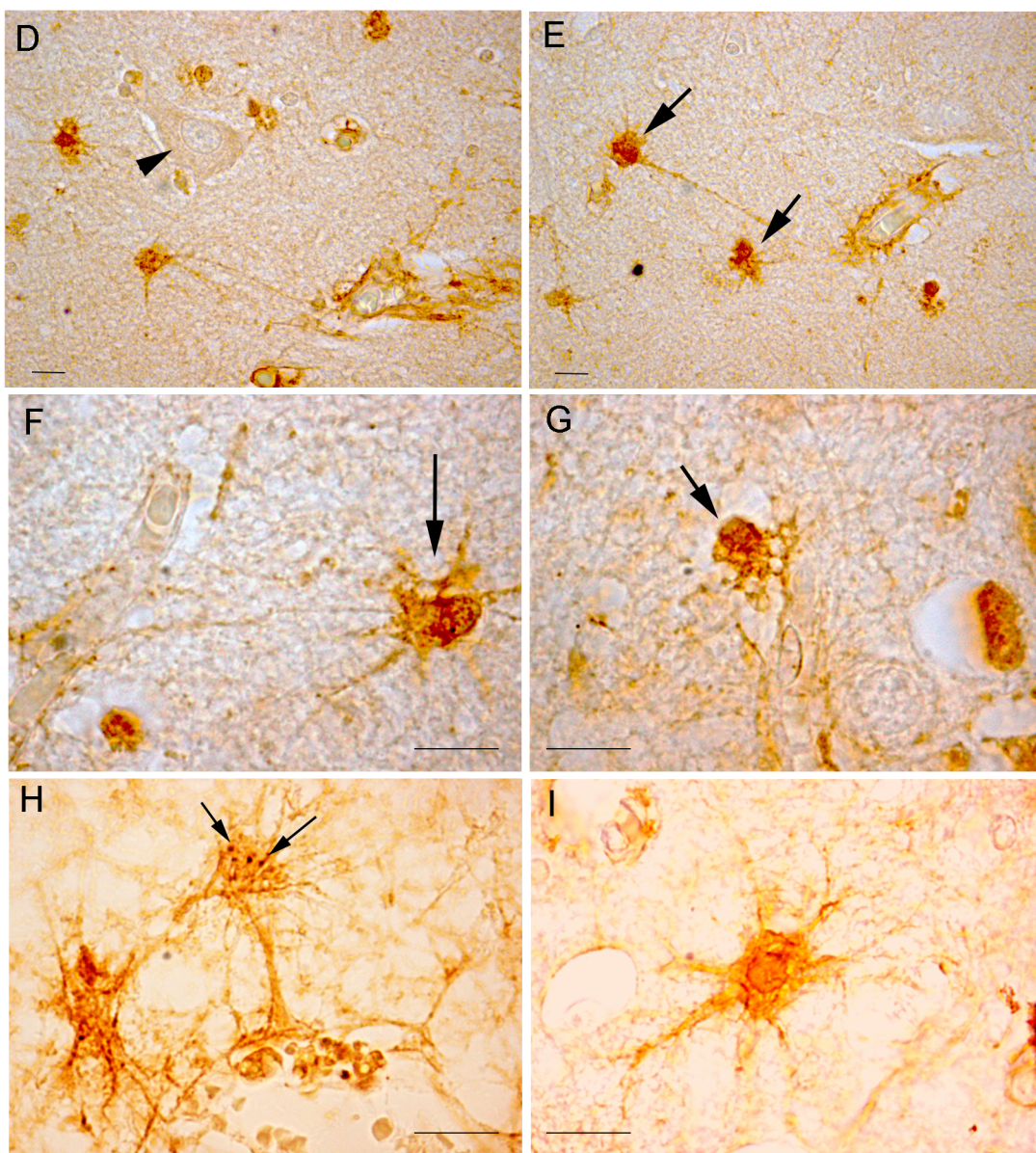

Supplement: Supplementary file 1 — Supplementary Figure 1: Control of immunolabelling and ACE2 expression in adult hippocampus. A-C) Pictures of the hippocampus fimbrial angle in control processed sections without primary ant-ACE2 antibodies: A) rabbit polyclonal anti-ACE2 (Abcam Cat# ab15348). Control sections processed with rabbit polyclonal anti-ACE2 (Sigma-Aldrich Cat#HPA000288) and mouse monoclonal anti-ACE2 (R&D Systems Cat# MAB933) were similar to this one (data not shown). B) rat monoclonal anti-GFAP (Millipore Cat# 345860-100UG). Control sections processed with goat polyclonal anti-Doublecortin (Santa Cruz Biotechnology Cat# sc-8066) were similar to this one (data not shown); C) rabbit polyclonal anti-TBR2 (Abcam Cat# ab23345). D-G) ACE2 expression has been detected in paraffine section of adult hippocampus, demonstrating specific expression of ACE2 (rabbit ati-ACE2 from Abcam) in astroglia in D, E and F (arrows), and pericytes in G (arrow). While ACE2-expressing cells are identified by brown-colored neurons were negative as we can observe in D by the white color (arrowhead). H) Double immunohistochemistry showing GFAP expression (brown) and ACE2 expression (black dots; arrows) in astroglial cells. I) Control section processed with GFAP and without ACE2 antibodies, showing the GFAP derived immunoreactivity and absence of ACE2 immunostaining. Scale bar: D-I) 50 µm [file 18_2023_4787_MOESM1_ESM.pdf]

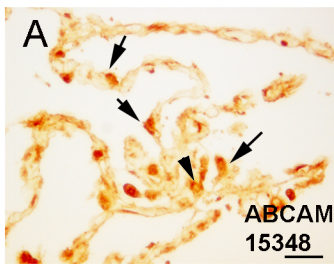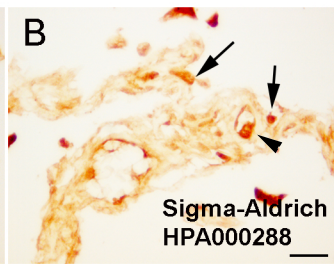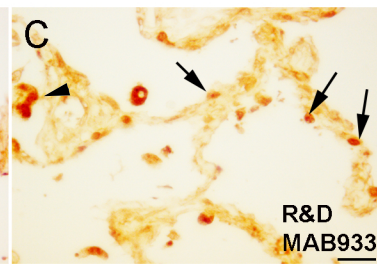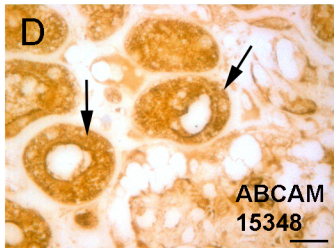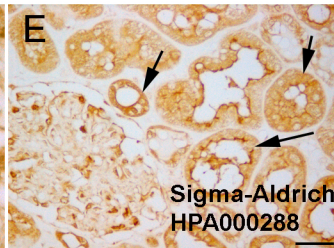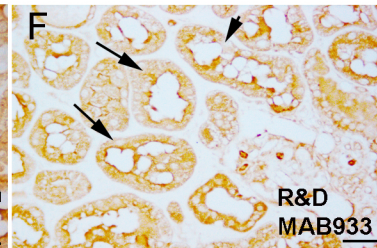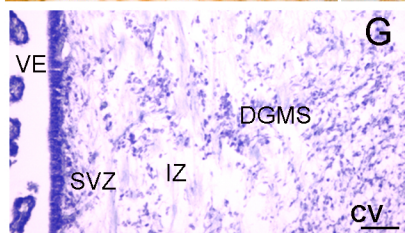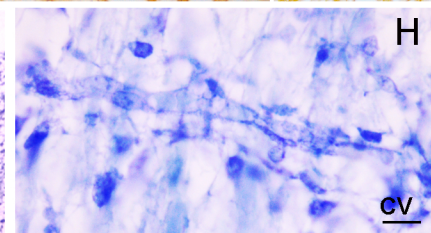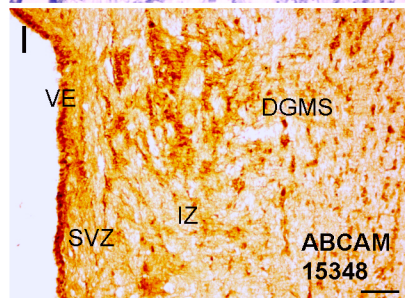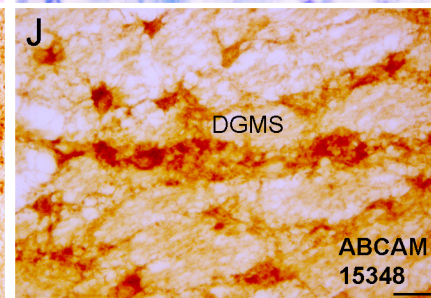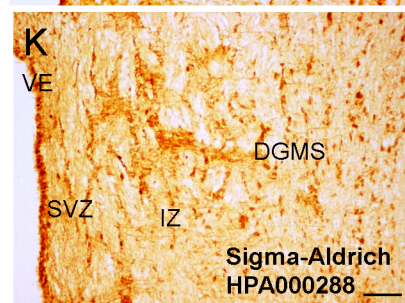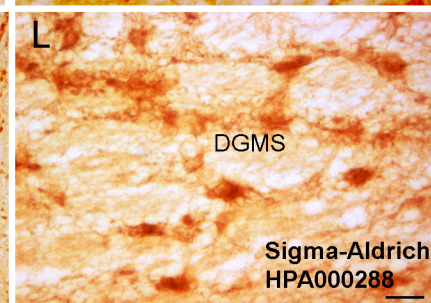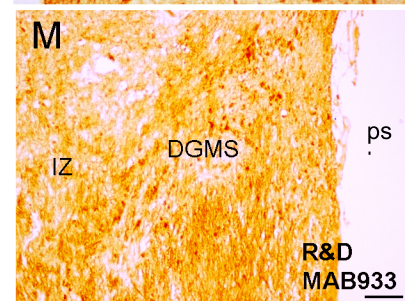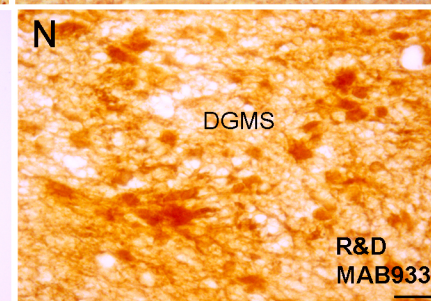

Supplement: Supplementary file 2 — Supplementary Figure 2 is not the revised version and has to be changed by the uploaded one, which contains the revised lettering: Specificity of ACE2 expression in human lung, kidney and developing brain. A-C) The three antibodies show ACE2-specific immunoreaction in human lung in alveolar endothelial type II cells (arrows) and capillary endothelium (arrowheads). D-F) The three antibodies show ACE2-specific immunoreaction in human renal proximal tubule cells (arrows). G) Low power picture of a brain section processed in parallel by immunohistochemistry without primary ACE2 Sigma-Aldrich antibody and counterstained with Cresyl violet. H) High power picture of a brain section processed in parallel by immunohistochemistry without primary ACE2 R&D antibody and counterstained with Cresyl violet. Control sections without primary antibodies do not showed immunostaining. I-N) The three antibodies show ACE2-specific immunoreaction in DG progenitors and migrating cells in DGMS. Scale bar: A, B, C) 50 µm; D, E, F) 25 µm; G, I, K, M) 100 µm; J, L, N) 25 µm. DGMS: Dentate Gyrus Migratory Stream; IZ: Intermediate Zone; SVZ: Subventricular zone: VE: Ventricular Epithelium [file 18_2023_4787_MOESM2_ESM.pdf]
